# Body Composition Changes Following a Concurrent Exercise Intervention in Perimenopausal Women: The FLAMENCO Project Randomized Controlled Trial

**DOI:** 10.3390/jcm8101678

**Published:** 2019-10-14

**Authors:** Irene Coll-Risco, Pedro Acosta-Manzano, Milkana Borges-Cosic, Daniel Camiletti-Moiron, Pilar Aranda, Alberto Soriano-Maldonado, Virginia A. Aparicio

**Affiliations:** 1 Department of Physiology, Faculty of Pharmacy, and Institute of Nutrition and Food Technology(INYTA), Biomedical Research Centre (CIBM), University of Granada, 18071 Granada, Spain; paranda@ugr.es (P.A.); virginiaparicio@ugr.es (V.A.A.); 2 Sport and Health University Research Institute (IMUDS), 18016 Granada, Spain; acostapedro23@gmail.com (P.A.-M.); milkana@ugr.es (M.B.-C.); 3 Department of Physical Education and Sports, Faculty of Sport Sciences, University of Granada, 18071 Granada, Spain; 4 Department of Physical Education, School of Education Sciences, University of Cádiz, 11519 Cádiz, Spain; daniel.camiletti@uca.es; 5 Biomedical Research and Innovation Institute of Cádiz (INiBICA) Research Unit, Puerta del Mar University Hospital University of Cádiz, 11009 Cádiz, Spain; 6 Department of Education, Faculty of Education Sciences, University of Almería, 04120 Almería, Spain; asoriano@ual.es; 7 SPORT Research Group (CTS-1024), CERNEP Research Center, University of Almería, 04120 Almería, Spain

**Keywords:** fat mass, bone mineral content, body weight, pharmaceutical costs, climacteric

## Abstract

We assessed the effects of a 16-week primary-care-based exercise program on body composition in perimenopausal women. The women (*n* = 150) were randomized into control (*n* = 75) or exercise (*n* = 75) groups. Exercise was provided in a 16-week (60 min/session, 3 days/week) concurrent program. Body composition was measured using dual-energy X-ray absorptiometry. These are secondary analyses of the FLAMENCO Project (Clinical Trials Reference NCT02358109). In the intention-to-treat analyses, the control group showed no changes in body mass index (BMI) between post- and pre-test, whereas the exercise group showed a 0.75 kg/m^2^ decrease in BMI (95% CI: −1.29 to −0.22; *p* = 0.006). Gynoid and android fat mass in control group decreased by 98.3 g and 46.1 g after the 16 weeks, whereas they decreased by 213 g and 139 g in the exercise group, respectively (95% CI: −209 to −3.86; *p* = 0.042 and 95% CI: −164 to −26.9; *p* = 0.007, respectively). The control group decreased their pelvis bone mineral content by 2.85 g in the post-test compared with the pre-test, whereas the exercise group increased it by 1.13 g (95% CI: 0.93 to 7.81; *p* = 0.013). Per-protocol analyses showed similar results. These analyses suggest that the exercise intervention decreased fat depositions and BMI. Exercise might improve bone mineral content in specific areas such as the pelvis.

## 1. Introduction

Menopause, which is a crucial period for women’s health due to hormonal changes [1,2], is frequently associated with weight gain and central body fat accumulation [3]. This abdominal obesity can contribute to the development of dyslipidemia and insulin resistance [4,5]. Consequently, menopause is associated with a higher incidence of cardiovascular diseases [1]. Adiposity indices, in addition to being indicators for obesity, are directly associated with the incidence of metabolic syndrome [6]. A related problem that occurs during the menopause transition is estrogen loss [2,7], which decreases bone mass [8], thus increasing the risk of bone fracture [9]. Therefore, improving body composition and preserving bone mass is a desirable goal in this physiological stage.

The effects of different types of exercise on body composition have been widely studied in pre- and post-menopausal women [1,10,11,12,13,14]. Some studies have shown improvements in some body composition variables as a result of high intensity training [1,10], moderate aerobic training [11], or resistance training [12,13]. Other studies failed to find positive changes regarding body weight [10] or fat measurements [14]. This controversy underlines that further research is needed to understand the best exercise modality to optimize body composition during this period.

Simultaneously, evidence exists that exercise can prevent bone loss and osteoporosis [15], and higher levels of muscle strength have been associated with greater bone mineral density (BMD) [16]. Combined resistance and high-impact (or weight bearing) training improved BMD and showed lower ratio of bone fracture [17], whereas resistance training alone showed no effects [17,18]. Some aerobic protocols have produced improvements in BMD [18]; however, others have been ineffective [19]. Although high-impact exercise has positive effects on bone health and aerobic exercise may improve lean and fat mass, a combination of aerobic and resistance training could simultaneously improve both body composition components. To the best of our knowledge, no prior study has explored the effects of a concurrent exercise program on body composition during perimenopause.

In addition to the above, the cardiovascular diseases and bone mass loss linked to menopause are associated with greater health services and drug prescription costs (e.g., medication for osteoporosis or hypertension) than those of the general population [20,21]. We hypothesized that the exercise intervention in this study may be associated with healthier body composition, and body composition is related to several of the diseases mentioned [1,4,6]. Therefore, our exercise program could reduce symptoms and rates of diseases that may lead to a reduction of pharmaceutical costs. Thus, it is of clinical and public health interest to examine whether positive changes in body composition are associated with lower pharmaceutical costs.

Therefore, the aims of this study were (a) to evaluate the effects of a 16-week concurrent exercise program on body composition compared with control intervention in perimenopausal women; and (b) to assess the association of body composition changes with pharmaceutical costs. 

These are secondary analyses from the FLAMENCO study randomized controlled trial [22]. Primary analyses for the FLAMENCO study have been previously published [23]. 

## 2. Materials and Methods

### 2.1. Participants

The complete methodology of the FLAMENCO study was published previously [22]. Briefly, inclusion criteria for the current randomized controlled trial were: (1) aged 45–60 years old; (2) not having severe somatic or psychiatric disorders. Exclusion criteria were: (1) acute or terminal illness; (2) having suffered a major cardiovascular event in the past 6 months; (3) unable to ambulate; (4) unstable cardiovascular disease or other medical condition; (5) fracture; (6) unwillingness to complete the study requirements; (7) presence of neuromuscular disease or drugs affecting neuromuscular function; (8) having an osteoarticular prosthesis.

In this randomized controlled trial, a total of 214 perimenopausal women (age 45–60 years old) from Granada (Southeast Spain) were recruited through primary care centers and press releases published in local newspapers and social media. All women provided informed consent to participate in the present study. 

### 2.2. Randomization

After checking inclusion criteria, a total of 150 women voluntarily participated and were randomized into either a control (*n* = 75) or exercise (*n* = 75) group. A computer-generated simple randomization sequence was created to allocate the participants to either the exercise or control group (1:1). The randomization sequence was prepared by a member of the research team with no clinical involvement in the trial.

### 2.3. Procedures

The same group of researchers recorded socio-demographic and clinical characteristics as well as body composition on a single day and in this order: socio-demographic (including age, educational, and marital status, among others), clinical, and other health-related information was collected using a self-reported questionnaire (quality of life, anxiety, depression, food frequency questionnaires, and fitness). This anamnesis also included questions regarding their clinical history. The use of hormone therapy or osteoporosis drugs was registered. 

Women also completed a Food Frequency Questionnaire [24], validated in an Andalusian population (the population to which the participants in the present study belong), which was employed to determinate the frequency of consumption of a list of 78 foods. Based on those results, the Mediterranean Diet Score [25] was calculated to evaluate the degree of adherence to the traditional Mediterranean diet, as well as the consumption of different groups of foods (i.e., non-refined cereals, potatoes, fruits, vegetables, legumes, fish, olive oil, red meat and derivates, poultry, full fat dairy products, and alcohol).

The Blatt–Kupperman menopausal index [26] was employed to assess menopause symptomatology. 

Cardiorespiratory fitness was assessed with the modified Bruce test [27,28], upper-body muscle strength with the handgrip strength test [29,30], and lower body muscle strength with the 30 s chair stand test [29]. 

This study was approved by the Ethics Committee of the Hospital Virgen de las Nieves (Granada, Spain). This trial was registered at ClinicalTrials.gov (identifier: NCT02358109).

Data collection was performed at baseline (within one week before the intervention) and after 16 weeks (i.e., after the intervention period).

### 2.4. Exercise Intervention

The 75 women among the exercise group were divided into 4 smaller groups, with 15–20 participants for each instructor. The allocation of the women in the different schedules (two in the morning, one in the afternoon, and one in the evening) only depended on their availability. The women randomized into the exercise group participated in a 16-week (60 min/session, 3 days/week) primary-care-based exercise intervention consisting of a moderately vigorous intensity concurrent exercise program (aerobic and resistance training). This exercise protocol was designed by an expert team of graduates in sports sciences, following the training standards recommendations by the American College of Sports Medicine for adults [31], which suggests involvement in some kind of moderate to vigorous physical activity for at least 150 min per week to produce substantial health benefits.

Each exercise session included a 10 min warm-up period with walking and mobility exercises, followed by the 40 min main part, which varied across week days (i.e., 3 different models of session). The sessions finished with a 10 min cool-down period of stretching and relaxation exercises. The first session of the week involved circuit training including resistance exercises in a stepped progression throughout the program. The second session of the week included balance-oriented activities (position changes, monopodal and bipodal stances, etc.) and dancing (aerobic exercises). The third session of the week combined aerobic, resistance, and coordination exercises within the same session [22]. Regardless of the women’s previous physical activity, we monitored the ratings of perceived exertion using the Borg 6–20 ratings of perceived exertion (RPE) [32] scale during all sessions. The intensity (expressed as RPE) ranged from 12 to 16, so that trainers could help personalize the training intensity for each woman. All trainings sessions were performed under the supervision of a team of personal trainers. 

### 2.5. Control Group 

The participants in the control group did not participate in the exercise sessions and they were requested to continue their daily activities. However, as a healthy diet and increasing physical activity levels and exercise have proven beneficial effects, participants in the control group attended four workshops (one per month) addressing different topics: (1) benefits of exercise for longevity, prevention, and treatment of diseases, lectured by a BSc in nutrition; (2) benefits of the Mediterranean diet and nutritional education; (3) ergonomic advises and exercises to perform at home (e.g., strength training); and (4) strategies to increase their daily physical activity levels; all lectured by specialists in the topic (BSc in sports sciences). We also used these conferences to maintain their participation and loyalty until the end of the program. For this reason, the control group could be considered a counselling intervention. We only invited the control group to these conferences.

### 2.6. Outcomes

#### 2.6.1. Anthropometry and Body Composition

A portable eight-polar tactile electrode impedanciometer (InBody R20, Biospace, Seoul, Korea) was used to measure body weight. Height (cm) was measured using a stadiometer (Seca 22, Hamburg, Germany). We measured lean mass, fat mass, visceral adipose tissue, gynoid fat mass, android fat mass, total BMD, BMD of lumbar spine and pelvis, total bone mineral content (BMC), and BMC of pelvis using a dual-energy X-ray absorptiometry (DEXA) device (Hologic Discovery QDR, Massachusetts, USA). We calculated body mass index (BMI) as weight (kg) divided squared height (m), as well as fat mass index (FMI) with the same formula: fat mass (kg) divided squared height (m).

#### 2.6.2. Pharmaceutical Costs

The pharmaceutical consumption of each patient was determined through the medical history from the DIRAYA system, used by the Public Health System of Andalusia [33]. 

We calculated the cost of medication with the 2015 prices in Spain. The consumption costs of prescribed pharmaceuticals for each patient before and during the study were calculated based on the prices, prescribed dose, and schedule of administration. More details regarding the complete methodology used to collect pharmaceutical costs was published previously [23]. From all the medications used by the women in the study, only those considered to influence or be influenced by body composition and exercise were registered. This included drugs for osteoporosis, anxiolytics, anti-inflammatories, analgesic, relaxants, anti-depressives, hormonal therapies, and thyroid drugs. 

To consider all possible medication changes over the 16-week intervention, data on medication in both groups were recorded at the initial, middle, and final phase of the study. 

#### 2.6.3. Physical Activity Levels 

A triaxial accelerometer GT3X+ (Actigraph, Pensacola, FL, USA) was used to measure activity counts (rate of 30 Hz and stored at an epoch length of 60 s). The women wore the device on the hip near their center of gravity, underneath clothing, and secured with an elastic belt.

Physical activity was measured the week before starting the exercise intervention and the week after finishing it, 16 weeks later. It was recorded for up to 9 days starting from the day that the women received the accelerometers until the day they were instructed to return the devices. The number of minutes of moderately vigorous physical activity bouts per week was also calculated. Bouted moderately vigorous physical activity was defined as a period of ≥10 consecutive minutes spent in that behavior. Women were classified on whether they met the American College of Sports Medicine guidelines for adults [31], (>150 min/week of bouted moderately vigorous physical activity) Data download, reduction, cleaning, and analyses were performed using ActiGraph software (ActiLife v. 6.11.9; Actigraph, Pensacola, FL, USA).

#### 2.6.4. Statistical Analysis

Descriptive statistics (mean (standard deviation, SD) for quantitative variables and number of women (%) for categorical variables) were employed to describe the baseline characteristics of the study sample. To detect potential differences on these outcomes between the groups, the following statistical tests were performed: independent sample Student’s *t*-test (normal distribution, homoscedasticity), Welch’s test (normal distribution, heteroscedasticity) and Mann–Whitney U test (non-normal distribution) for continuous variables, and the Chi-square test for categorical variables.

According to the original protocol [22], the effects of a 16-week exercise intervention on lean, fat, and bone mass were assessed using linear regression in an intention-to-treat [ITT] (main analysis) and per-protocol basis. In those participants with missing data at follow-up, values were estimated using a mean imputation procedure (Table 2). We included the changes (post–pre) in body composition as dependent variables in separate models and the group (control = 0 and exercise = 1) as independent variable. Baseline outcome values were controlled by including them as covariables in all models. For weight, BMI, and lean and fat mass variables, we tested the influence of some potential confounders related to diet (i.e., alcohol consumption, meat, fish, eggs, pulses, cereals, dairy products, fats, vegetables, fruits, sweets, beverages, and nuts) as we could not assess energy intake directly with bivariate correlation. As the results remained unchanged after adjusting for the different food groups, we did not include them in the final models. As for bone-related variables, after considering different confounders based on previous literature, age, fat and lean mass, and hormone therapy were considered and finally included as potential confounders, as the between-group differences changed importantly (i.e., >10%) after their inclusion. We considered meeting the minimum physical activity recommendations as well as the change between the post- and pre-test in the number of minutes of moderate to vigorous activity as a new confounders, but the results remained the same, and we did not include them. 

Per-protocol analyses, including only those participants with ≥75% attendance to the intervention, were also carried out to assess the clinical efficacy of the exercise program.

Linear regression analyses were used to assess the association of changes in body composition parameters (independent variable) with changes in pharmaceutical cost (dependent variable) (Table S1).

The statistical analysis was conducted with the Statistical Package for Social Sciences (IBM SPSS Statistics for Windows, Version 20.0, Armonk, NY, USA). The statistical significance was set at *p* < 0.05.

## 3. Results

**Figure 1:**
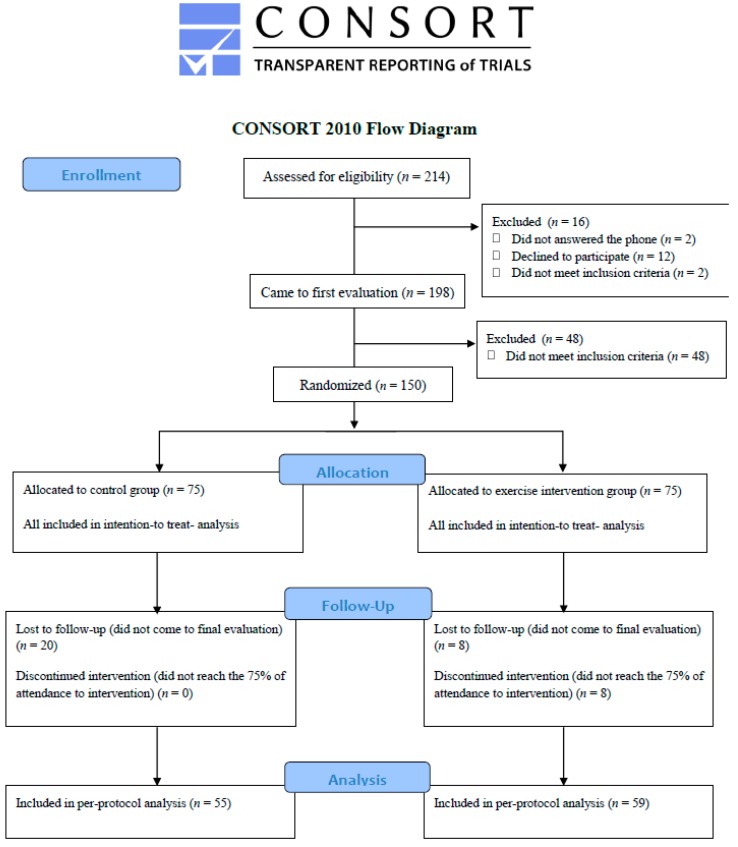
Flow chart of the study participants.

Of the 150 women that were randomized to control (*n* = 75) and exercise (*n* = 75) groups used on the intention-to-treat (primary analyses), 20 and 8 of them were lost to follow-up in the control and exercise groups, respectively. A total of eight women did not attend 75% of the exercise sessions. Thus, the total number of women used for per-protocol analyses was 114 divided into control (*n* = 55) and exercise (*n* = 59) groups. A flowchart of the study participants is shown in Figure 1.

The baseline characteristics of the study participants by group are listed in Table 1. The control and exercise groups scored means of 15.0 and 16.6 points, respectively, in the Blatt–Kupperman index assessed at baseline, being mildly affected by menopause [26]. No differences between the control and exercise groups were found in any of the sociodemographic and clinical variables at baseline (all *p* > 0.05, Table 1). 

Table 2 shows the ITT analyses of body composition changes between pre- and post-intervention for control and exercise groups. The control group decreased their body weight by 0.07 g in the post-test compared with the pre-test, whereas the exercise group decreased by 1.52 g; the exercise group decreased 1.45 g more than control group (between group differences: 95% CI: −3.32 to 0.39; *p* = 0.121). The BMI of the control group did not change in the post-test compared with the pretest, whereas the BMI of the exercise group decreased 0.75 kg/m^2^; the exercise group decreased 0.75 kg/m^2^ more than control group (between group differences: 95% CI: −1.29 to −0.22; *p* = 0.006). Gynoid fat mass and android fat mass in control group decreased by 98.3 g and 46.1 g in the post-test compared with the pre-test, whereas they decreased by 213 g and 139 g in the exercise group, respectively; the exercise group decreased 115 g and 92.9 g more than control group in gynoid fat mass (between-group differences: 95% CI: −209 to −3.86; *p* = 0.042) and in android fat mass (between-group difference: 95% CI: −164 to −26.9; *p* = 0.007), respectively.

The control group decreased their pelvis BMC by 2.85 g in the post-test compared with the pretest, whereas the exercise group increased it by 1.13 g; the exercise group increased the pelvis BMC by 3.98 g more than control group (between group differences: 95% CI: 0.93 to 7.81; *p* = 0.013). No differences between groups were observed for lean mas, fat mass, visceral adipose tissue, total BMD, total BMC, or pelvis and lumbar spine BMD. 

The per-protocol analysis (Table 3) showed similar results in BMI, android fat mass, and BMC of pelvis, but no differences were found in gynoid fat mass. The control group decreased their total BMC by 32.9 g in the post-test compared to the pretest, whereas the exercise group decreased it by 9.38 g; the control group reduced their total BMC by 23.5 g more than the exercise group (between-group difference: 95% CI: 0.38 to 69.0; *p* = 0.048). In per-protocol analyses, the control group increased their pharmaceutical expenditure by €13.6/month in the post-test compared with the pretest, whereas the exercise group decreased it by €2.01/month; the exercise group reduced their pharmaceutical expenditures by €15.7/month more than the exercise group (between-group difference: 95% CI: −27.5 to −2.77; *p* = 0.017).

We performed an exploratory analysis to study the association between changes in body composition (observed in the ITT analyses), and changes in pharmaceutical costs (Table S1). We observed that improvements in BMI and pelvis BMC after the intervention showed no association with reduced pharmaceutical expenditures (B = 2.05, β = 0.10, 95% CI: −1.29 to 5.39, *p* = 0.228; B = −0.24, β = 0.08, 95% CI: −0.74 to 0.27, *p* = 0.360, respectively). However, improvements in gynoid fat mass after the exercise program was associated with reduced pharmaceutical costs (B = 0.02; β = 0.23; 95% CI: 0.01 to 0.04; *p* = 0.006). Similarly, improvements in android fat mass were associated with a reduction in pharmaceutical expenditures (B = 0.03; β = 0.21; 95% CI: 0.01 to 0.06; *p* = 0.011).

## 4. Discussion

The main findings of these secondary analyses of the FLAMENCO Project suggest that a 16-week concurrent exercise program decreases BMI and gynoid and android fat mass and increases BMC of the pelvis, compared with a control group that received health counselling intervention. Per-protocol analyses further revealed a smaller reduction in total BMC in the exercise group compared to the control group, suggesting a potential clinical efficacy of exercise to reduce total BMC loss. However, we observed no effects on other relevant markers of adiposity such as total fat mass, FMI, fat mass percentage, or visceral adipose tissue. The reductions found in gynoid and android fat mass were associated with lower pharmacological expenditure. 

Other similar studies reported comparable results [1,10,11,12,14,34,35]. Although body weight showed a meaningful reduction in the exercise group, we found no significant differences between groups after the 16-week intervention. Similarly, Maillard et al. [10] found no differences in body weight in either the high-intensity or moderate-intensity 16-week programs compared to baseline [10]. Contrary to these findings, the women in the study of Arsenault et al. [11] showed a significant reduction in body weight after an exercise program, three to four times per week at a moderate intensity over six months. Di Blasio et al. [12] also found weight differences between control and exercise groups after a 13-week low-intensity program. These controversial results might suggest the need for a large trial to understand the effects of different exercise intensities and types of training on weight loss in this population. The greater positive effects of dietary plus exercise interventions on body composition compared with uniquely dietary or exercise-based programs should be considered, especially regarding weight loss and adiposity [36,37]. Notwithstanding, we aimed to isolate the exercise effect to explore the influence of this type of concurrent training on body composition in this specific population.

Despite the lack of clear significant differences in weight loss, we found a significant reduction in BMI in the exercise compared with the control group. These results are meaningful as BMI is known to be a useful tool for assessing overweight and obesity [38], and menopause is associated with increased weight status and, consequently, a higher risk of cardiovascular diseases [1]. 

Although we expected a clear lean mass increase in the exercise group, the results showed no significant differences between groups. It is possible that the healthy lifestyle conferences provided to the control group during the intervention period might have highly motivated these women to lead a more active lifestyle. The lack of results could also be explained by missing energy intake balance. Some data suggest that exercise programs result in a compensatory reduction in physical activity levels throughout the rest of the day [34,39]. This, together with the minimal differences found between groups of women meeting the minimum recommendations for physical activity, might partially explain the weak improvements in body weight and fat mass in the exercise group. This hypothesis might, therefore, explain the various null results observed in this study. Lean mass increased less than expected in the exercise group. As a previous study observed a greater increase in lean mass following a high intensity [1] program, it is possible that the intensity or the length of the exercise program in this study was insufficient to significantly increase lean mass.

Regarding fat mass, our moderately vigorous program resulted in gynoid and android fat mass reductions in the exercise compared with the control group, but no other fat measurements showed a significant reduction. Similar to our results, Grossman and Payne [14] did not find any reductions in fat mass measurements in either same-length short-duration high-intensity exercise or in their moderate exercise groups when compared to baseline [14]. Bouchonville et al. [35] found no changes in visceral mass or overall body weight after one year of concurrent exercise. However, they found differences, in line with what was previously stated [36,37], when exercise was combined with dietary intervention [35], indicating the high influence of diet on this kind of interventions. Conversely, Mandrup et al. [1] found changes in all fat variables studied after a three-month high-intensity training program compared to baseline. Maillard et al. [10] found differences in total fat mass in both high-intensity and moderate-intensity programs compared to baseline, but only high-intensity interval training produced a reduction in abdominal and visceral fat. Therefore, higher intensity interval training programs should be considered for future projects, as well as the inclusion of dietary programs for better results.

The exercise group showed no differences in total BMD or lumbar spine or pelvis BMD compared with the control group. An increase in pelvis BMC was found in the exercise group. A healthier profile in total BMC in the exercise compared to the control group was also confirmed when conducting the per-protocol analyses. Despite this last result not being confirmed in the intention-to-treat analysis, it suggests a potential clinical efficacy of the exercise program to reduce total body BMC loss. Another study [17] suggested that resistance training combined with high-impact training improved BMD in the spine and femoral neck, while resistance training alone showed no differences [17]. Following this results, Heinonen et al. [18] found no differences in BMD after resistance training. However, our concurrent exercise program resulted in no changes in lumbar spine BMD. Heinonen et al. [18] also found that an 18-month program based on endurance training produced a significant trend indicating the maintenance of BMD in the distal radius and femoral neck. Conversely, Wen et al. [19] found no differences between the exercise and control group in BMD after a 10-week program based on step aerobic exercise. During perimenopausal years, bone loss accelerates and even with exercise (especially regarding muscle strength), it may be impossible to load bones enough to prevent or retard bone loss during this critical transition period [16,40]. Therefore, we might state that the length of the program could be the reason for the minimal changes observed in bone in the present study, as changes in BMC and mostly BMD are long term. 

Notably, we also observed that a reduction in gynoid and android fat mass, as seen after our exercise program in the intention-to-treat analysis, impacted pharmaceutical cost savings, and hence health care costs. No association was observed between either the improved BMI or the pelvis BMC with a reduced pharmaceutical expenditure. However, when analyzing with per-protocol data, a between-group difference was associated with the overall pharmaceutical cost, which showed a meaningful reduction in the expenditure in medication for women that followed the training program. We are aware that such a short period of time prevents obtaining strong evidence in pharmaceutical costs. However, we expected to observe some short-term influence of the exercise training program on the consumption of anxiolytics, anti-inflammatories, analgesic, and relaxants. Therefore, this minor expense in medication costs might probably be increased in the long term (more time for the physician to adjust pharmacology prescription after having more information regarding the evolution of the diseases plus more time to improve body composition through exercise). 

Despite the drop-out rate of women from the control and exercise groups, and the discontinued intervention of eight women, we decided to present the data as intention-to-treat analyses, being potentially important as they replicate how this kind of program would work in real life. Per-protocol analyses were also added, being able to isolate and evaluate the clinical efficacy of the present concurrent exercise intervention. Future studies are warranted to confirm or contrast what type of exercise programs might benefit perimenopausal women’s body composition while promoting cost savings for health systems. Several studies analyzed the association of different diseases, such as cancer [41] or osteoporosis [42], with pharmaceutical costs, or analyzed the costs of a specific drug on a similar population to ours. However, as far as we know, the relationship between changes in body composition and pharmaceutical costs has not been addressed. We provide novel and useful data and our findings should increase interest in finding exercise programs that can promote improvements in body composition with parallel reductions in pharmaceutical costs. This should also lead to future studies with a larger sample size to verify the present results. 

### Strengths and Limitations

Firstly, the women included in this study were not particularly affected by menopause (mild severity) and showed moderate–high physical activity levels, which hinder the extrapolation to women severely affected by menopause, or with low physical activity levels. As a result, these findings might not be generalizable to other populations of perimenopausal women with higher rates of BMD loss. Secondly, this study lacks serum hormonal analyses to objectively assess menopause status (e.g., estrogens, follicle stimulating hormone, and luteinizing hormone). Thirdly, the assessment of energy intake would have helped to better understand the effects of the exercise program on body composition parameters, and should be considered in future studies. The Food Frequency Questionnaire used in this study is a qualitative tool that cannot be used to measure energy intake, and other questionnaires recording quantity of servings would be preferable for future studies. However, we performed different sensitivity analyses with different food group consumptions and the results remained unchanged. More specific bone analyses (i.e., vertebral and femoral neck specific scanners) would have added relevant information to the current study. Finally, we did not measure physical activity levels during the intervention but only before and after, and we could not follow the potential changes in physical activity along the program, which would have provided valuable data. In exercise trials, the participants often reduce their daily physical activity levels because they follow the exercise program [34]. 

We evaluated many outcomes, and some of the associations may be due to chance. Thus, caution is advised in the interpretation of the results and further research to confirm or contrast these results is warranted. However, the measurement tool employed to assess body composition (i.e., DEXA) is widely valid and reliable, which guarantees the quality of these data. 

## 5. Conclusions

The main findings of these secondary analyses of the FLAMENCO Project suggest that a 16-week concurrent exercise program decreases BMI and gynoid and android fat mass and increases BMC of the pelvis, compared with a control group that received health counselling intervention. Per-protocol analyses further revealed a smaller reduction in total BMC in the exercise group compared to the control group, suggesting a potential clinical efficacy of exercise to reduce total BMC loss. However, we observed no effects on other relevant markers of adiposity such as total fat mass, FMI, fat mass percentage, or visceral adipose tissue. The reductions found in gynoid and android fat mass were associated with lower pharmacological expenditure. Considering these results, finding a specific training program that may optimize improvements in body composition and may reduce pharmaceutical expenditur appears necessary. This kind of result has no precedents and is of clinical and public health importance. 

## Figures and Tables

**Table 1:** Baseline characteristics of the study participants.

	Control Group	Exercise Group	
	Mean (SD)	Mean (SD)	*p*
	(*n* = 75)	(*n* = 75)	
Age (years)	52.7 (4.5)	52.8 (4.5)	0.877
Height (cm)	159.2 (5.8)	159.7 (6.0)	0.492
Sedentary time (mins/week)	3293.0 (525)	3421.1 (728)	0.344
Light PA (mins/week)	2968.6 (576)	2962.7 (596)	0.751
Moderately vigorous PA (mins/week)	180.8 (145.5)	178.6 (157.2)	0.759
Women meeting PA recommendations * pre-test (%)	54.5	45.5	0.383
Mediterranean Diet score pre-test	31.1 (5.1)	31.1 (3.9)	0.610
Mediterranean Diet Score post-test	33.3 (0.6)	33.7 (0.5)	0.403
Kupperman global score (0–45)	15.0 (10.2)	16.7 (10.8)	0.456
**Fitness at pre-test**			
VO_2max_ (mL/kg/min)	21.3 (5.3)	19.6 (4.7)	0.064
Strength legs (repetitions)	15.2 (2.2)	15.5 (2.3)	0.526
Handgrip (kg)	27.0 (4.2)	25.8 (3.9)	0.113
**Medicines/supplements**			
Women taking hormone treatment (thyroid) (%)	4.0	2.7	0.620
Women taking vitamin D (%)	2.7	0.0	0.224
Women taking calcium carbonate, yes (%)	9.4	2.7	0.135
Women taking zoledronic acid (%)	2.7	1.3	0.654
**Baseline body composition**			
Weight (kg)	70.1 (12.0)	69.7 (12.8)	0.616
Body Mass Index (kg/m^2^)	27.6 (4.2)	27.5 (4.9)	0.523
Lean mass (kg)	37.7 (4.8)	37.4 (4.9)	0.534
Fat mass (kg)	29.4 (7.6)	29.2 (7.9)	0.689
Fat Mass Index (kg/m^2^)	11.6 (2.9)	11.3 (2.8)	0.543
Fat mass percentage, %	42.0 (4.9)	41.9 (4.8)	0.862
Visceral adipose tissue (kg)	0.71 (0.3)	0.66 (0.3)	0.175
Gynoid fat mass (kg)	5.1 (6.3)	5.2 (1.3)	0.589
Android fat mass (kg)	2.5 (0.9)	2.5 (0.9)	0.538
Total bone mineral density (g/cm^2^)	1.14 (0.1)	1.15 (0.1)	0.329
Total bone mineral content (g)	2117.2 (286)	2157.2 (294)	0.297
Bone mineral content of pelvis (g)	185.8 (43.7)	191.3 (43.6)	0.316
Bone mineral density of pelvis (g/cm^2^)	1.12 (0.1)	1.12 (0.1)	0.829
Bone mineral density of lumbar spine (g/cm^2^)	0.88 (0.1)	0.89 (0.1)	0.831
**Costs of medication**			
Monthly pharmaceutical expenditure pre-test (€)	20.1 (38.0)	27.7 (54.9)	0.245
Monthly pharmaceutical expenditure post-test (€)	32.4 (72.4)	30.7 (90.7)	0.299

SD, Standard Deviation; PA, Physical activity; €, euro; * Minimum recommendations for this population are 150 min/week of moderate to vigorous physical activity. *p*-values were calculated using independent sample Student’s *t*-test (normal distribution, homoscedasticity), Welch’s test (normal distribution, heteroscedasticity), and Mann–Whitney U test (non-normal distribution) for continuous variables, and the Chi-square test for categorical variables.

**Table 2:** Intention-to-treat analyses showing the effect of a 16-week concurrent exercise program on body composition in perimenopausal women (*n*=150).

*Body Composition*	Control (*n* = 75)	Exercise (*n* = 75)	Between-Group Difference (95% CI)	*p*
	Change from Baseline at Week 16 (SD)	Change from Baseline at Week 16 (SD)		
Weight * (kg)	−0.07 (3.48)	−1.52 (6.30)	−1.45 (−3.32 to 0.39)	0.121
Body Mass Index * (kg/m^2^)	0.00 (1.34)	−0.75 (1.96)	−0.75 (−1.29 to −0.22)	0.006
Lean mass * (g)	471 (1280)	314 (1416)	−157 (−592 to 284)	0.489
Fat mass * (g)	−544 (1081)	−903 (2159)	−359 (−903 to 175)	0.184
Fat Mass Index (kg/m^2^)	−0.29 (0.48)	−0.41 (0.88)	−0.13 (−0.39 to 1.30)	0.325
Fat mass percentage * (%)	−0.72 (1.24)	−0.95 (2.20)	−0.23 (−0.80 to 0.32)	0.396
Visceral adipose tissue * (g)	−21.3 (67.0)	−30.5 (88.3)	−9.2 (−37.1 to 12.1)	0.315
Gynoid fat mass * (g)	−98.3 (221)	−213 (414)	−115 (−209 to −3.86)	0.042
Android fat mass * (g)	−46.1 (152)	−139 (268)	−92.9 (−164 to −26.9)	0.007
Total bone mineral density ^a^ (g/cm^2^)	−0.01 (0.02)	0.00 (0.02)	0.01 (−0.00 to 0.02)	0.114
Total bone mineral content ^a^ (g)	−23.6 (104)	−8.65 (34.5)	15.0 (−6.20 to 43.69)	0.140
Bone mineral content of pelvis ^a^ (g)	−2.85 (10.2)	1.13 (11.5)	3.98 (0.93 to 7.81)	0.013
Bone mineral density of pelvis ^a^ (g/cm^2^)	−0.03 (0.03)	0.00 (0.03)	0.03 (−0.00 to 0.02)	0.171
Bone mineral density of lumbar spine ^a^ (g/cm^2^)	−0.01 (0.07)	0.00 (0,08)	0.01 (−0.01 to 0.03)	0.319
Pharmaceutical expenditure per woman * (€/month)	12.3 (34.4)	2.95 (35.8)	−9.35 (−19.4 to 3.22)	0.160

Note: Mean results show the differences between post-pre intervention results for each variable. ITT, Intention-to-treat analyses; SD, Standard Deviation; €, euro. * Model adjusted by baseline value of the variable. ^a^ Model adjusted by baseline value of the variable, age, height, and fat and lean mass.

**Table 3:** Per-protocol analyses showing the effect of a 16-week concurrent exercise program on body composition in perimenopausal women (*n* = 114).

*Body Composition*	Control (*n* = 55) Differences from Baseline to 16 Weeks (SD)	Exercise (*n* = 59) Differences from Baseline to 16 Weeks (SD)	Between-Group Difference (95% CI)	*p*
Weight * (kg)	−0.01 (3.49)	−1.69 (6.65)	−1.68 (−3.18 to 0.23)	0.090
Body Mass Index * (kg/m^2^)	0.03 (1.61)	−0.91 (2.16)	−0.79 (−1.46 to −0.12)	0.022
Lean mass * (g)	655 (1474)	344 (1478)	−311 (−841 to 288)	0.334
Fat mass * (g)	−758 (1213)	−1063 (2257)	−305 (−984 to 426)	0.435
Fat Mass Index (kg/m^2^)	−0.41 (1.46)	−0.44 (1.05)	−0.08 (−4.83 to 3.21)	0.692
Fat mass percentage * (%)	−1.00 (1.37)	−1.12 (2.26)	−0.02 (−0.82 to 0.60)	0.743
Visceral adipose tissue * (g)	−29.7 (77.6)	−32.6 (92.0)	−2.9 (−37.8 to 27.6)	0.759
Gynoid fat mass * (g)	−136 (250)	−242 (435)	−106 (−213 to 55.1)	0.245
Android fat mass * (g)	−64.2 (176)	−162 (283)	−97.8 (−197 to −18.5)	0.018
Total bone mineral density (g/cm^2^)	−0.01 (0.02)	0.00 (0.02)	0.01 (0.00 to 0.01)	0.110
Total bone mineral content ^a^ (g)	−32.9 (121)	−9.38 (34.9)	23.5 (0.38 to 69.0)	0.048
Bone mineral content of pelvis ^a^ (g)	−3.97 (11.9)	1.51 (12.2)	5.48 (0.67 to 9.70)	0.025
Bone mineral density of pelvis ^a^ (g/cm^2^)	0.00 (0.03)	0.01 (0.03)	0.01 (−0.01 to 0.01)	0.734
Bone mineral density of lumbar spine ^a^ (g/cm^2^)	−0.01 (0.08)	0.00 (0.08)	0.01 (−0.01 to 0.05)	0.200
Pharmaceutical expenditure per woman * (€/month)	13.6 (34.5)	−2.01 (28.3)	−15.7 (−27.5 to −2.77)	0.017

Note: Mean results show the differences between post-pre intervention results for each variable. SD, Standard Deviation; €, euro. * Model adjusted by baseline value of the variable. ^a^ Model adjusted by baseline value of the variable, age, height, and fat and lean mass. Only women with available data and participants in the exercise group who attended ≥75% of the exercise sessions were included.

## Supplementary Materials

The following are available online at https://www.mdpi.com/2077-0383/8/10/1678/s1, Table S1: Association between the change in body composition and changes in pharmaceutical expenses.
