# Supplementary material for: Body Composition Changes Following a Concurrent Exercise Intervention in Perimenopausal Women: The FLAMENCO Project Randomized Controlled Trial
Source: J Clin Med. 2019 Oct 14;8(10):1678. doi: 10.3390/jcm8101678 (PMC6832269; doi:10.3390/jcm8101678)
Supplement: Supplementary file 1 [file jcm-08-01678-s001.pdf]

Table S1. Association between the change in body composition and changes in pharmaceutical expenses.

	Unstandarized Coefficients	Standarized Coefficients		Confidence interval 95% (B)	
	B	β	P	Lower	Upper
Body Mass Index (kg/m^2)	2.05	0.10	0.228	-1.29	5.39
Gynoid fat mass (g)	0.02	0.23	0.006	0.01	0.04
Android fat mass (g)	0.03	0.21	0.011	0.01	0.06
Bone mineral content of pelvis (g)	-0.24	0.08	0.360	-0.74	0.27
